# Heterogeneity of astrocyte density, morphology and connexins in the mouse hippocampus

**DOI:** 10.3389/fnana.2026.1771439

**Published:** 2026-03-09

**Authors:** Annika Uelwer, Mamitha Sivakumar, Khojimurod Umirdinov, Fathima Faiba A. Purath, Lensa Oluma, Max Anstötz, Charlotte von Gall, Amira A. H. Ali

**Affiliations:** 1 Faculty of Medicine, Institute of Anatomy II, Heinrich Heine University, Düsseldorf, Germany; 2 Department of Human Anatomy and Embryology, Faculty of Medicine, Mansoura University, Mansoura, Egypt

**Keywords:** astrocyte morphology, connexin, gap junctions, GFAP, spatial heterogeneity

## Abstract

The hippocampal formation is crucial for episodic learning and memory. In addition to neurons, astrocytes have also received increasing attention in recent years as essential components of brain networks by regulating the blood-brain barrier, eliminating waste products via the glymphatic system, supporting neuronal activity by providing energy supply and metabolic substrates, and regulating extracellular neurotransmitter levels. Astrocytes are heterogeneous and highly dynamic cells that respond to neuronal activity and dysfunction via morphological and functional changes. Astrocytic connexins (Cx) 30 and 43 form the molecular basis for gap junctions and hemichannels and are, thus, central to coupling, intercellular communication and network integration of astrocytes in the brain. However, little is known about the spatial heterogeneity of astrocyte density, morphology and Cx expression in the subregions and layers of the hippocampus. Therefore, in this study, we used immunohistochemistry to analyze the density and detailed morphological features of astrocytes and the spatial distribution of Cx30 and Cx43 in the layers of CA1, CA3 and dentate gyrus (DG). Astrocyte density correlated positively with the intensity of Cx30- and Cx43-immunoreaction (Ir). The stratum lacunosum moleculare (SLM) of CA1 and CA3 and the subgranular zone (SGZ) of DG showed the highest density of GFAP-positive (+) astrocytes and the strongest Cx30- and Cx43-Ir. The GFAP+ astrocytic processes had the largest radial extent in the pyramidal layer of CA1 and CA3 and in the granular layer of the DG. Our study provides a comprehensive anatomical and comparative mapping of astrocytic density, morphology and Cx distribution in the mouse hippocampus and provides an important basis for further studies on the dynamics of neuron-glial interaction under different physiological and pathological conditions.

## Graphical Abstract

**Figure:**
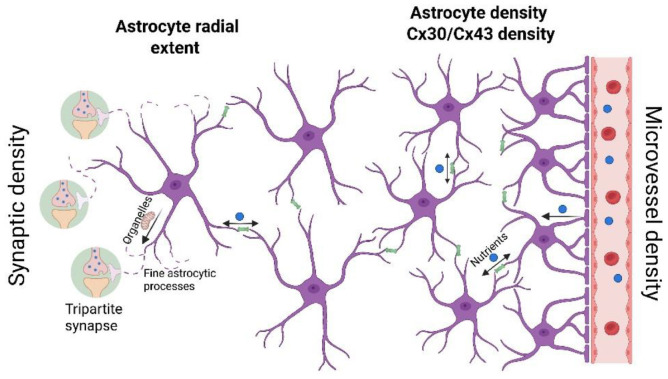
Schematic illustration showing the spatial diversity of astrocytes where the highest density of GFAP+ astrocytes, the smallest radial extension of GFAP+ processes, and the highest density of Cx30 and Cx43 are found in the subregions where the most microvessels exist and, thus, are correlated to blood-brain barrier (BBB) function and higher metabolic activity of astrocytes in these layers. The largest radial extension of GFAP+ processes is found in subregions that have the highest synaptic density (Created by Biorender.com).

## 1 Introduction

Elucidating the complex hippocampal network is pivotal for understanding cognitive processes, especially in the context of neurological diseases such as Alzheimer’s disease and temporal lobe epilepsy. The anatomy of the hippocampus in laboratory rodents such as mice and rats does not appear to differ significantly from that of primates and humans in terms of neural connections and layers (Zhao and Palomero-Gallagher, 2025; An et al., 2014).

The hippocampal formation is crucial for episodic learning and memory. It consists of the entorhinal cortex (EC), the subiculum (Sub), the dentate gyrus (DG) and the cornu ammonis (CA). The CA is divided into three distinct fields, CA1, CA2, and CA3, based on the cytoarchitecture of the pyramidal cells. In addition to preprocessed sensory information (Manns et al., 2007; Aronov et al., 2017), the hippocampal formation also integrates information about social interactions (Hitti and Siegelbaum, 2014; Lopez-Rojas et al., 2022), as well as aversive and appetitive stimuli (Skelin et al., 2019) and subcortical afferents (Amaral and Cowan, 1980; Vertes et al., 2004) that convey alertness, mood, and emotions. The neural connection between the EC and the hippocampus appears to be important for encoding the preprocessed sensory information (Manns et al., 2007; Aronov et al., 2017), in particular, in a spatial and temporal context (Moser et al., 2017; Wood et al., 2000; Manns et al., 2007; Eichenbaum, 2013; MacDonald et al., 2011; Kraus et al., 2015; Aronov et al., 2017), essential for navigation. The classical concept initially assumed a tri-synaptic connection, in which preprocessed sensory information from the EC reaches the DG via the perforant path and from there via the mossy fibers to the CA3 and from there via the Schaffer collaterals to the CA1, from where the output is transmitted via the Sub. This concept has since been greatly expanded, and several parallel entorhinal-hippocampal loops are postulated, in which the medial EC (MEC) is more involved in the representation of spatial and temporal information, while the lateral EC (LEC) plays a greater role in the processing of object representations (Save and Sargolini, 2017; Moser et al., 2017) and social memory (Lopez-Rojas et al., 2022).

DG and CA have a trilaminar structure; a cell-rich layer with excitatory principal neurons, a polymorphic layer with the axons of the principal neurons, and a cell-poor layer with the dendrite trees of the principal neurons and afferent fibers. In the DG, these layers are referred to as granule cell layer (Gr), polymorphic layer (Pol) and molecular layer (Mol), the latter containing mainly the perforant path. Between Gr and Pol is a narrow layer of cells, the subgranular zone (SGZ), which is an important site of adult neurogenesis, where neuronal stem cells and progenitor cells proliferate, differentiate and produce new granule cells. In CA1, the first two layers are called the pyramidal cell layer and stratum oriens (Or), the latter of which also contains the basal dendrites of the pyramidal cells. According to their afferent connections, the cell-poor layer of the CA, in which the apical dendrites of the pyramidal cells are located, is divided into the proximal stratum radiatum (Rad) and the stratum lacunosum moleculare (SLM) adjacent to the hippocampal fissure (Zhao and Palomero-Gallagher, 2025; Valero and de la Prida, 2018). Or and Rad receive inputs from Schaffer collaterals as well as from subcortical telencephalic structures such as septal nuclei. Afferents from the entorhinal area and the thalamus also end in the Or and in the SLM. Afferents from the brainstem, on the other hand, end mainly in the proximal part of the SLM.

In addition to neurons, which have been the subject of intensive research for a long time, astrocytes have also been receiving increased attention in recent years as essential components of brain networks. They significantly contribute to maintain brain function by regulating the blood-brain barrier, acting as a perivascular sheath for lymphatic drainage, and thus waste clearance, and playing a crucial role in neuron metabolism by supporting energy supply, providing metabolic substrates, and regulating the extracellular environment including the redox balance (Almeida et al., 2023) and the level of ions and neurotransmitters (Bélanger et al., 2011). The latter function also plays an important role in ensuring efficient synaptic transmission, as astrocytes take up and recycle neurotransmitters, which is why the unit consisting of neurons and astrocytes is also referred to as a tripartite synapse. In particular, the uptake of glutamate by astrocytes is of special importance, as this prevents neuronal excitotoxicity. In addition, astrocytes modulate neuronal activity by releasing gliotransmitters such as ATP, glutamate, and D-serine. Therefore, an intact astrocytic network is vital for neural homeostasis, plasticity and cognition. Within the hippocampus, astrocytes seem to regulate stress and anxiety-like behaviors (Li et al., 2024), via modulation of astrocytic glucocorticoid receptors (Lu et al., 2025). In addition, the astrocytic calcium activity is correlated with the affective state and plays an important role in anxiolytic behavior due to enhanced ATP release (Cho et al., 2022). Furthermore, astrocytic G-protein-coupled receptors critically regulate the working and spatial memory (Escalada et al., 2024). Accordingly, their dysfunction is associated with various neurological disorders, including Alzheimer’s disease, temporal lobe epilepsy and anxiety-related disorders.

The complex and diverse functions of astrocytes are also reflected in their complex structure. They have a tree-like morphology with processes that envelop the blood vessels on the one hand and the synapses on the other. Astrocytes express connexons, hexameric protein complexes composed of six connexin (Cx) subunits, in particular Cx30 and Cx43 (Huang et al., 2021), that form hemichannels in the plasma membranes (Goodenough and Paul, 2009). Hemichannels allow the release of gliotransmitters such as glutamate, ATP, and aspartate into the extracellular space, thus facilitating communication between astrocytes and neighboring neurons (Ye et al., 2003; Xing et al., 2019). Two connexons from neighboring astrocytes form a gap junction that allows the passage of ions, small metabolites, and signaling molecules, resulting in electrical and metabolic coupling and, thus, a functional syncytium in the astrocytic network (Dossi et al., 2024; Kielian, 2008). Astrocytic Cx are essential for synaptic efficacy (Cheung et al., 2023; Pannasch et al., 2014), modulate neuronal network activity (Pannasch et al., 2011, 2014), contribute to neuroprotection, the integrity of blood-brain barrier (Ezan et al., 2012) and the glymphatic system (Cibelli et al., 2021) and might also be involved in adult neurogenesis (Talaverón et al., 2024). Our previous study shows that Cx30 and Cx43 play a role in the stability of circadian rhythms (Ali et al., 2019). In various neurodegenerative diseases and neuroinflammation, changes in astrocytic Cx can be observed, which in turn have an effect on progression by altering neuronal function and survival (Huang et al., 2021; Kielian, 2008). Astrocyte-specific Cx30 and Cx43 double knockout (Cx30/43 DKO) mice show changes in circadian rhythm stability (Ali et al., 2019), as well as deficits in sensorimotor performance and a complete lack of spatial learning and memory (Hösli et al., 2022). The latter indicates the special role of the astrocytic Cx for the hippocampal network. In fact, excitatory synaptic transmission and long-term potentiation of CA1 neurons are significantly affected in Cx30/43 DKO mice (Hösli et al., 2022).

The glial fibrillary acidic protein (GFAP) is an intermediate filament (IF) type III protein, a structural marker that is found in the majority of CNS astrocytes (Jurga et al., 2021). The density and morphology of astrocytes in the cerebral cortex, including the hippocampus, shows layer-specific heterogeneity, which appears to be caused mainly by the different input signals (Viana et al., 2023; Karpf et al., 2022; Lanjakornsiripan et al., 2018). Cx also appear to exhibit region-specific differences in the brain (Nagy et al., 1999; Batter et al., 1992), which may be related to the activity level of surrounding neurons (Huang et al., 2019). However, little is known about the spatial heterogeneity of Cx in the sublayers of the hippocampus. Therefore, in this study, we analyzed the GFAP+ astrocyte density and the detailed morphological features as well as the spatial distribution of Cx30 and Cx43 in the sublayers of the CA and the DG using immunohistochemistry and sholl-based tracing. Our study contributes to detailed anatomical knowledge and better understanding of the heterogeneity of astrocyte morphology, density and subtypes as well as the formation of astrocytic gap junctions in relation to their neuronal environment, which could help probing the role of hippocampal astrocytes in learning, memory and anxiety modulation. This provides a basis for understanding the plasticity of astrocytes under physiological and pathological conditions.

## 2 Materials and methods

### 2.1 Animals and tissue processing

Adult male 12–15 weeks old C57BL/6J mice were housed in standard cages in temperature- and sound-proof cabinets (Beast master, Germany) with a light intensity of 600 lx during the light phase and 0 lx during the dark phase. Animal experiments were approved by the local government LANUV (North Rhine-Westphalia State Agency for Nature, Environment and Consumer Protection; Germany; AZ: 84–02.04.2013.A358) and conform to European and international guidelines. Mice were deeply anesthetized using intraperitoneal injection of ketamine-xylazine mixture (100 mg/10 mg per kg, respectively). This was followed by transcardial perfusion with 0.9% NaCl, then 4% formalin. After post-fixation in 4% formalin for additional 24 h, the brains were cryoprotected in 20% then 30% sucrose until saturation. Parallel series of 30 μm thick coronal brain slices containing the dorsal hippocampus, bregma from −1.58 mm to −2.18 mm (Paxinos and Franklin, 2012), were prepared using a cryomicrotome (Reichert-Jung).

### 2.2 Immunohistochemistry

For each primary antibody, one of the parallel series was used and stained in a free-floating manner. Slices were washed using phosphate-buffered saline (PBS) containing 0.2% Triton X-100 (PBST). To inhibit endogenous peroxidase activity, the slices were treated with 0.24% hydrogen peroxide (H_2_O_2_) for 30 min. Following this, the slices were washed and then incubated with 5% normal goat serum in PBST for one hour to block nonspecific binding. Sections were incubated overnight at 4 °C with one of following primary antibodies: polyclonal rabbit anti-glial fibrillary acidic protein (GFAP) (1:3000, cat#: Z0334, DAKO, Denmark), polyclonal rabbit anti-Cx30 (1:1500, cat#: 71–2200, Thermo Fisher), polyclonal rabbit anti-Cx43 (1:250, cat#:71–0700, Thermo Fisher). Specificity of the antibodies against Cx30 and Cx43 has been previously validated using Cx30/43 DKO mice (Ali et al., 2019). The sections were then washed with PBST and incubated with biotin-conjugated goat anti-rabbit IgG (1:500, Vector Laboratories, BA-1000) for one hour. This was followed by washing and then incubation with avidin-biotin-peroxidase complex (Vectastain ABC kit, Vector Laboratories) for one hour. Afterward, sections were rinsed in PBST followed by a 10-min incubation with 3,3′-diaminobenzidine (Sigma-Aldrich) and rinsed with PBS. The slices were mounted on slides, rinsed with water, dehydrated and coverslipped using Entellan (Merck Millipore).

### 2.3 Image acquisition and analysis

The following layers were analyzed in the CA1 and CA3: stratum oriens (Or), stratum pyramidale (Pyr), stratum radiatum (Rad), and stratum lacunosum moleculare (SLM). The following layers were analyzed in the DG: stratum polymorphe (Pol), molecular layer (Mol), stratum granulare (Gr), and the subgranular zone (SGZ) (Viana et al., 2023; Karpf et al., 2022; Paxinos and Franklin, 2012). The SGZ was defined as a two-nucleus-wide area along the inner border of Gr (Ali et al., 2015; Supplementary Figure 1).

Astrocytes were manually counted in a delineated area in each hippocampal subregion using ImageJ and astrocyte density was defined as the number of cells per mm^2^. For the analysis of astrocyte morphology, three GFAP-stained astrocytes per layer were selected per mouse for further analysis based on the following inclusion criteria: clear boundaries, intact and no or few overlapping processes with neighboring astrocytes, as previously described (Ali et al., 2020). Due to the extensive overlap of the processes, we could not include the SGZ in the morphological analysis. Z-stacks of images were taken using the 100× objective lens in bright-field microscope (Nikon Eclipse E800). The GFAP-stained astrocytes were traced and three-dimensionally reconstructed by Neurolucida software (version 4.3.3, MicroBrightField Inc.). The following parameters were then further analyzed by Sholl analysis and averaged per mouse: cell body surface area, mean terminal distance of the endings from the cell body to quantify the mean radial extent, total length of the processes, total number of branches, total number of trees (indicated by number of first order processes), nodes/100 μm length, and total surface area of the processes.

For Cx30 and Cx43 analyses, three images per mouse were taken in bright-field microscope (BZ-9000, Keyence, Japan) with identical settings e.g., light intensity and exposure time for each antibody. For semi-quantitative analysis, the staining intensity of Cx30 and Cx43-immunoreaction (Ir) was measured in the delineated layers using ImageJ software (NIH). The background intensity, determined in a cell-free area within the corpus callosum, was subtracted. The resulting Ir intensity was averaged per mouse and expressed in arbitrary units (au). Normalization of Cx-Ir to GFAP+ astrocyte density or area was not feasible, as staining sets for Cx and GFAP was performed using different cohorts of mice.

### 2.4 Statistical analysis

GraphPad Prism Software (GraphPad Software, Inc.) was used for statistical analysis. Normal distribution within groups was confirmed by using the D’Agostino and Pearson test. Data were presented as mean ± SEM. To assess differences between the subregions one-way ANOVA followed by Tukey’s *post-hoc* test for multiple comparison was used. To analyze the association between different parameters, the correlation coefficient (r) of pairs of variables from each hippocampal subregion was analyzed by Pearson Test. *P* < 0.05 was considered statistically significant.

## 3 Results

### 3.1 Heterogeneity of GFAP+ astrocyte density in hippocampal layers

**FIGURE 1:**
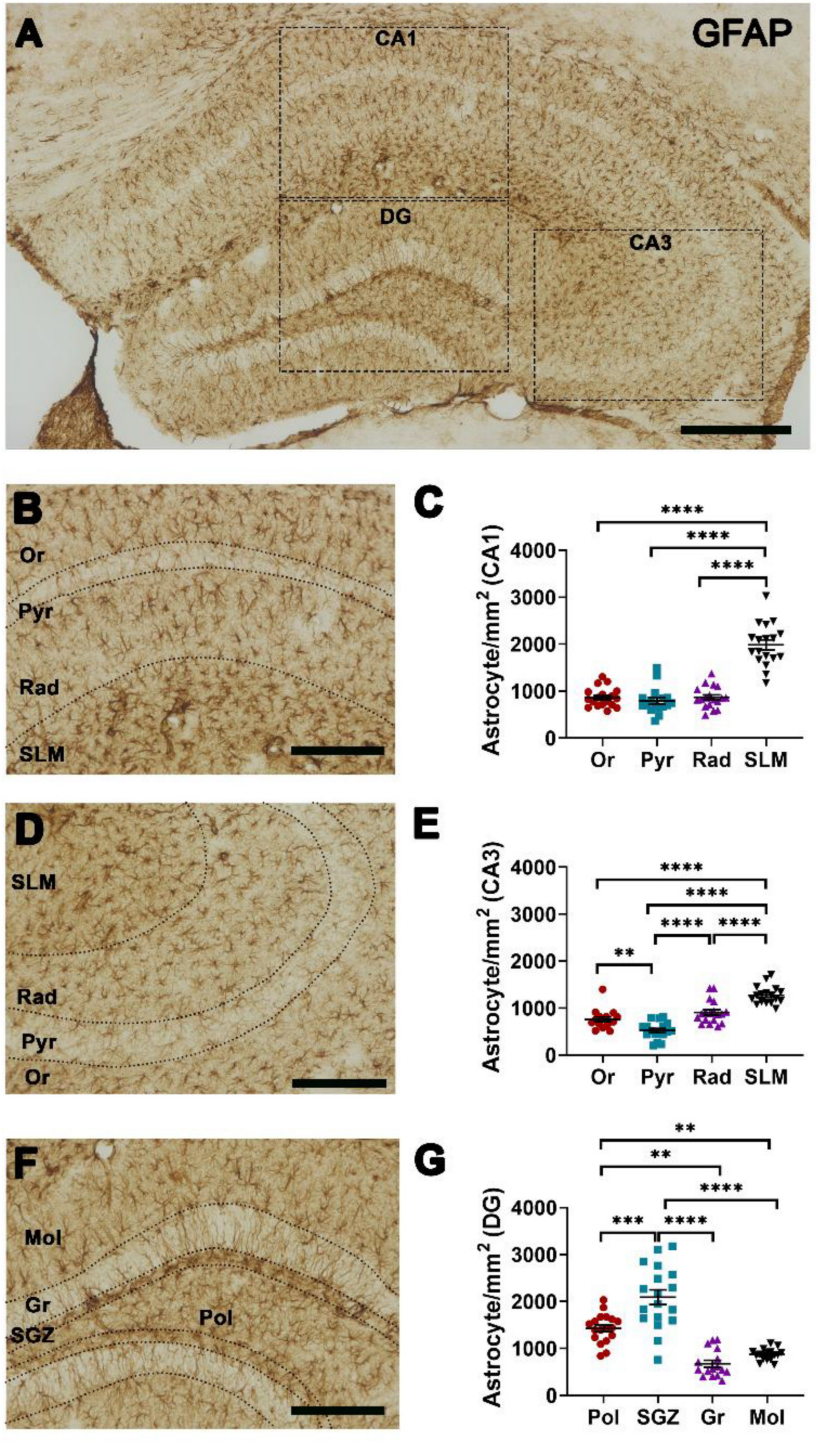
Heterogeneity in the density of GFAP-positive (+) astrocytes in the hippocampal layers. **(A)** Representative photomicrograph showing GFAP+ astrocytes in hippocampal CA1, CA3, dentate gyrus (DG) (insets), scale bar = 200 μm. Representative photomicrograph of GFAP+ astrocytes at higher magnification in **(B)** CA1 and **(D)** CA3 subregions: Or, stratum oriens; Pyr, stratum pyramidale; Rad, stratum radiatum; SLM, stratum lacunosum moleculare, scale bar = 100 μm. Quantification of GFAP+ astrocyte density in **(C)** CA1, **(E)** CA3. **(F)** Representative photomicrograph of GFAP+ astrocytes at higher magnification in DG subregions: Mol, stratum moleculare of DG; Gr, stratum granulare; SGZ, subgranular zone; Pol, stratum polymorphe, scale bar = 100 μm. **(G)** Quantification of GFAP+ astrocytes in DG. One-wayANOVA followed by Tukey *post-hoc* tests; **p < 0.01; ***p < 0.001, ****p < 0.0001. *n* = 18 mice.

The density of GFAP+ astrocytes was heterogeneous across the layers of hippocampal regions (Figure 1A). The highest astrocyte density in CA1 was found in the SLM, whereas there were no statistical differences among Or, Pyr and Rad (Figures 1B, C). In CA3, the SLM showed the highest astrocyte density, whereas Pyr showed the lowest astrocyte density (Figures 1D, E). Within DG, the highest astrocyte density was found in SGZ, whereas the lowest astrocyte density was found in Gr (Figures 1F, G).

### 3.2 Heterogeneity of GFAP+ astrocyte morphology in hippocampal layers

**FIGURE 2:**
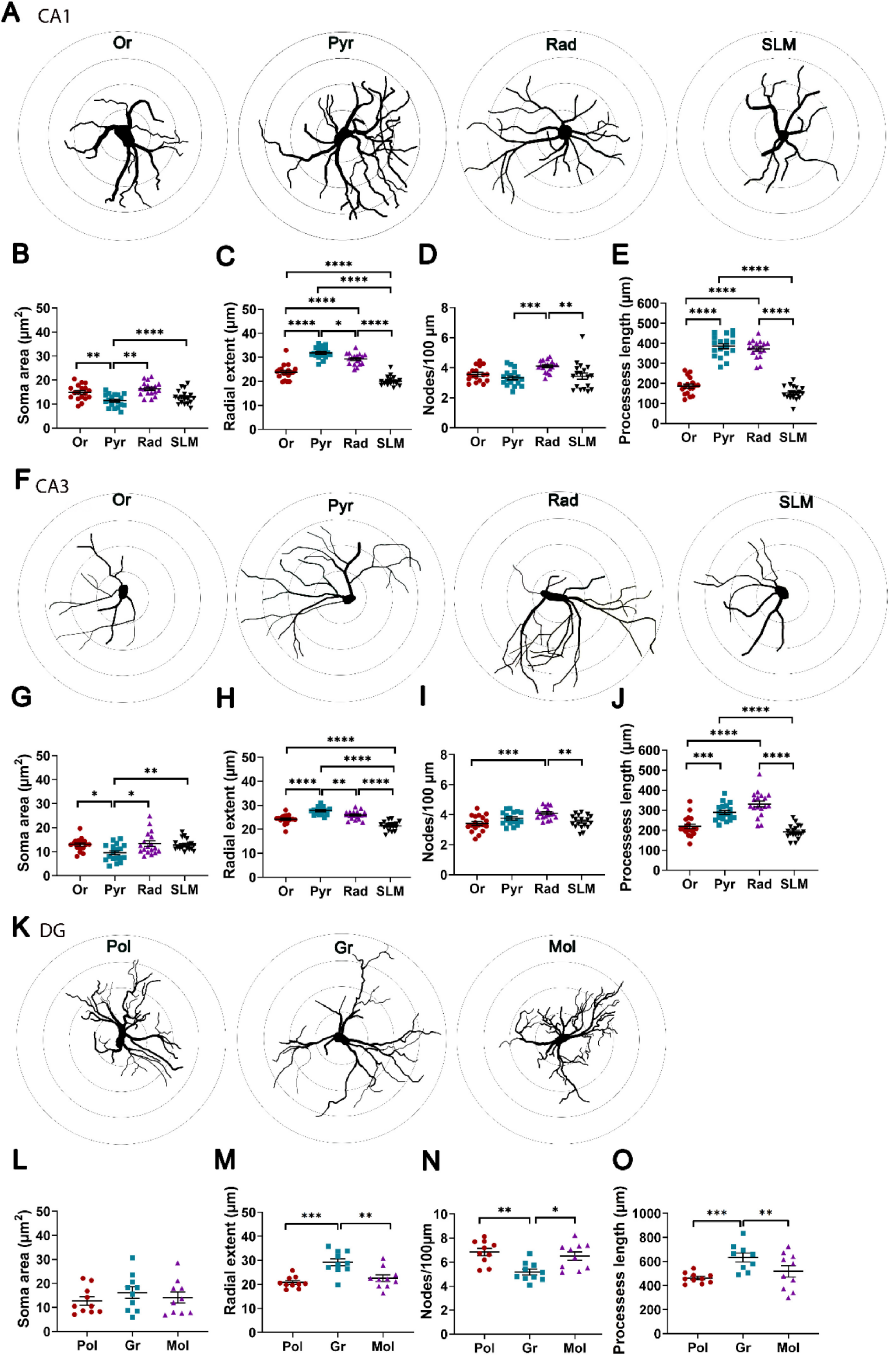
Heterogeneity in the morphology of GFAP-positive astrocytes in the hippocampal layers. **(A)** Representative Sholl-based tracing of astrocytes in CA1 layers in 10 μm segments from the soma center. **(B–E)** Quantification of astrocyte morphology variables in CA1. **(F)** Representative Sholl-based tracing of GFAP-positive astrocytes in CA3 layers. **(G–J)** Quantification of astrocyte morphology variables in CA3. **(K)** Representative Sholl-based tracing of GFAP-positive astrocytes in DG layers. **(L–O)** Quantification of astrocyte morphology variables in DG. Gr, stratum granulare; Mol, stratum moleculare of DG; Or, stratum oriens; Pol, stratum polymorphe; Pyr, stratum pyramidale; Rad, stratum radiatum; SGZ, subgranular zone; SLM, stratum lacunosum moleculare of CA1 and CA3. One-way ANOVA followed by Tukey *post-hoc* tests; *p < 0.05; **p < 0.01; ***p < 0.001, ****p < 0.0001 using. *n* = 10 in DG and *n* = 18 mice in CA1 and CA3.

Soma and processes of GFAP+ astrocytes showed morphological heterogeneity across the layers of CA1, CA3 and DG (Figure 2 and Supplementary Figure 2). In CA1 (Figures 2A, B) and CA3 (Figures 2F, G), the cell bodies were smallest in Pyr. The mean distance from the soma to the terminal point of the traced trees, as a measure for radial extent and total processes length, were largest in Pyr and shortest in SLM of both CA1 (Figures 2A, C, E) and CA3 (Figures 2F, H, J). The frequency of nodes/100 μm length, as a measure for the complexity of branching, was higher in Rad compared to Pyr and SLM in CA1 (Figures 2A, D) and to Or and SLM in CA3 (Figures 2F, I). In DG, the size of cell bodies was not different among Pol, Gr and Mol. However, in the Gr the GFAP+ processes showed the largest radial extent and the lowest complexity of branching (Figures 2K–O).

### 3.3 Heterogeneity of Cx30 and Cx43 immunoreaction in hippocampal layers

**FIGURE 3:**
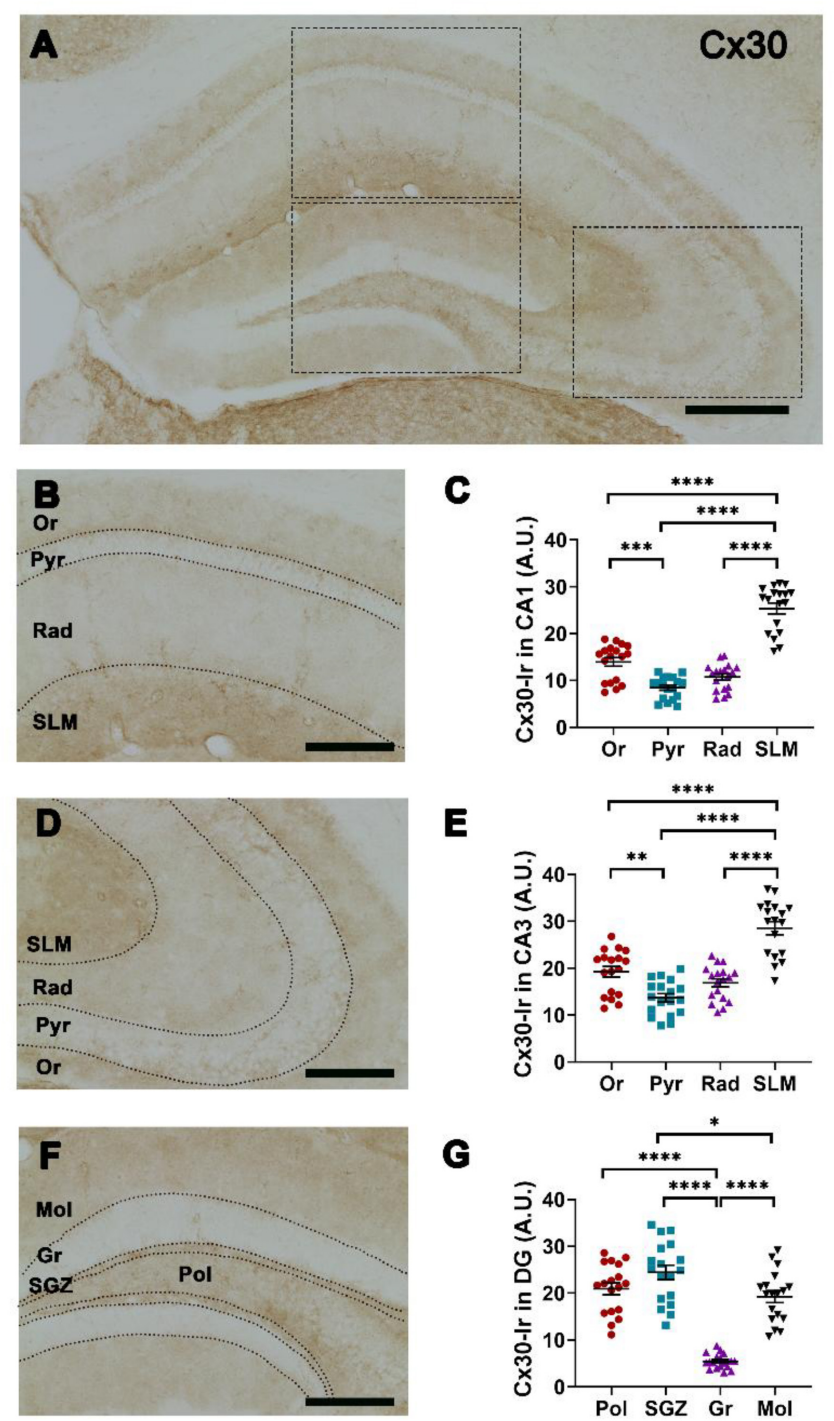
Spatial distribution of connexin 30 (Cx30) in hippocampal layers. **(A)** Representative photomicrograph showing Cx30-immunoreaction (Ir) in hippocampal CA1, CA3, dentate gyrus (DG) (insets), scale bar = 200 μm. Representative photomicrograph of Cx30-Ir at higher magnification in **(B)** CA1 and **(D)** CA3 subregions: Or, stratum oriens; Pyr, stratum pyramidale; Rad, stratum radiatum; SLM, stratum lacunosum moleculare, scale bar = 100 μm. Quantification of Cx30-Ir in **(C)** CA1, **(E)** CA3. **(F)** Representative photomicrograph of Cx30-Ir at higher magnification in DG subregions: Mol, stratum moleculare of DG; Gr, stratum granulare; SGZ, subgranular zone; Pol, stratum polymorphe, scale bar = 100 μm. **(G)** Quantification of Cx30-Ir in DG. One-way-ANOVA followed by Tukey *post-hoc* tests; *p < 0.05; **p < 0.01; ***p < 0.001, ****p < 0.0001. *n* = 18 mice.

Cx30-Ir showed the highest intensity in SLM and the lowest intensity in Pyr and Rad in both CA1 (Figures 3A–C) and CA3 (Figures 3A, D, E). Cx30-Ir was more intense in Or compared to Pyr in both CA1 (Figures 3A–C) and CA3 (Figures 3A, D, E). In DG, Cx30-Ir showed the highest intensity in SGZ and the lowest intensity in Gr, and lower intensity in Mol compared to SGZ (Figures 3A, F, G).

**FIGURE 4:**
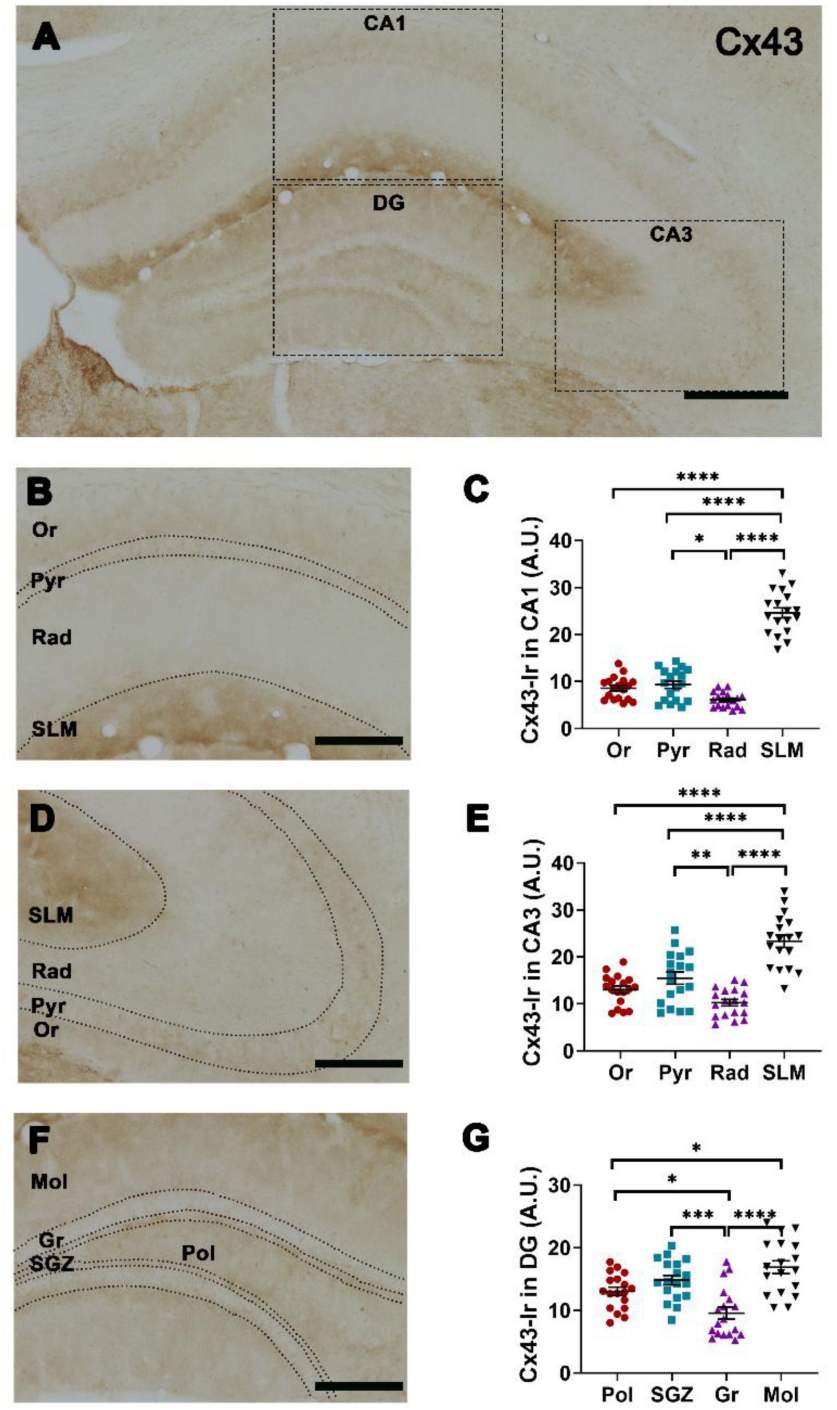
Spatial distribution of connexin 43 (Cx43) in hippocampal layers. **(A)** Representative photomicrograph showing Cx43-immunoreaction (Ir) in hippocampal CA1, CA3, dentate gyrus (DG) (insets), scale bar = 200 μm. Representative photomicrograph of Cx43-Ir at higher magnification in **(B)** CA1 and **(D)** CA3 subregions: Or, stratum oriens; Pyr, stratum pyramidale; Rad, stratum radiatum; SLM, stratum lacunosum moleculare, scale bar = 100 μm. Quantification of Cx43-Ir in **(C)** CA1, **(E)** CA3. **(F)** Representative photomicrograph of Cx43-Ir at higher magnification in DG subregions: Mol, stratum moleculare of DG; Gr, stratum granulare; SGZ, subgranular zone; Pol, stratum polymorphe, scale bar = 100 μm. **(G)** Quantification of Cx43-Ir in DG. One-way ANOVA followed by Tukey *post-hoc* tests; *p < 0.05; **p < 0.01; ***p < 0.001, ****p < 0.0001. *n* = 18 mice.

Cx43-Ir showed the highest intensity in SLM and the lowest intensity in Rad of both CA1 (Figures 4A–C) and CA3 (Figures 4A–E). In Pyr of both CA1 (Figures 4A–C) and CA3 (Figures 4A, D, E), Cx43-Ir was less intense than in SLM but more intense than in Rad. In DG, Cx43-Ir showed the highest intensity in Mol and the lowest intensity in Gr. Cx43-Ir intensity was significantly lower in Pol than in Mol (Figures 4A, F, G).

### 3.4 Correlation between Cx30, Cx43, GFAP+ astrocyte density and morphology

**FIGURE 5:**
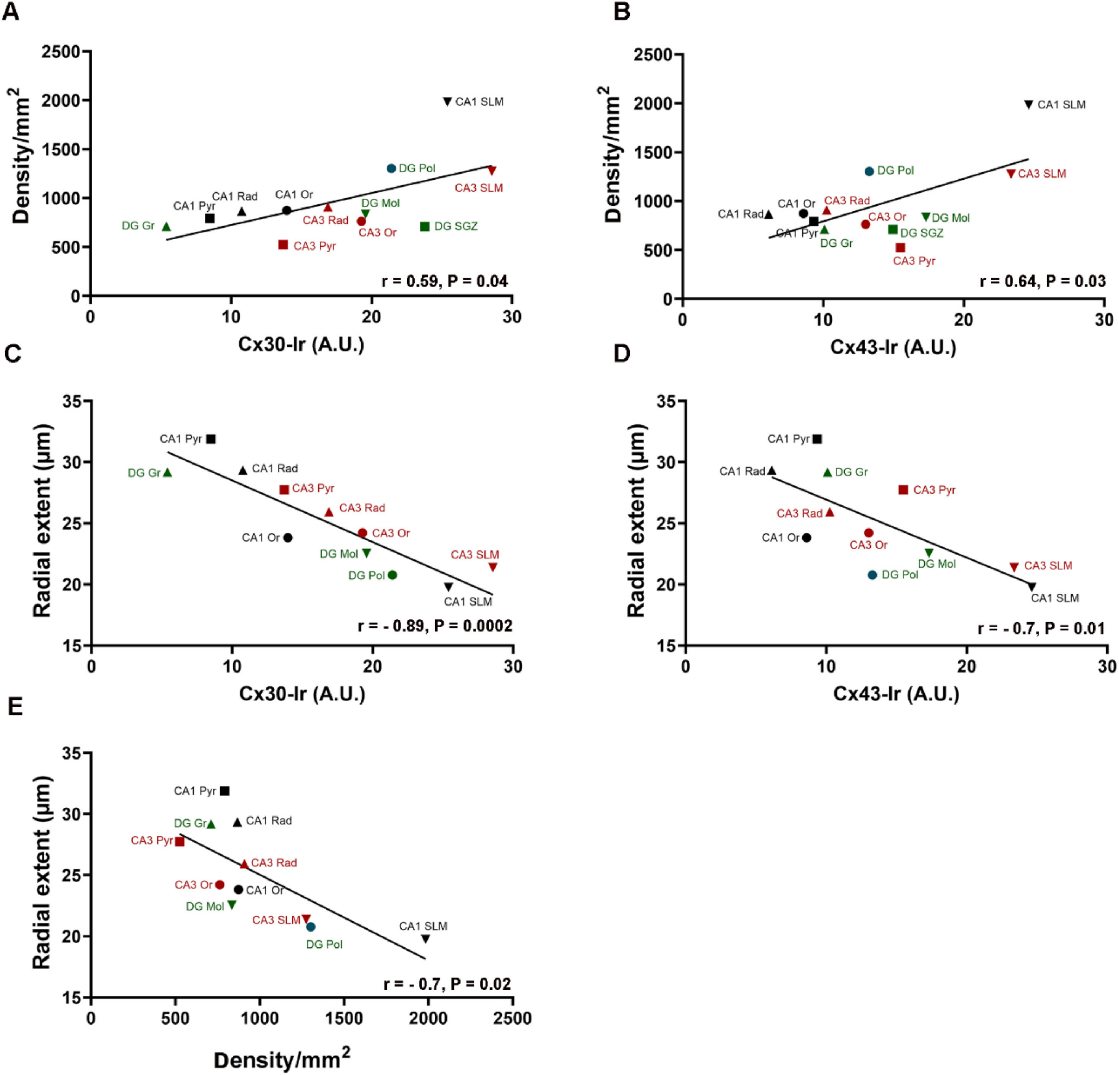
Correlation between connexins (Cx) and density and radial extent of GFAP positive (+) astrocytes in hippocampal layers. Scatterplots showing the relationship between density of GFAP+ astrocytes and **(A)** Cx30 and **(B)** Cx43, between the radial extent of GFAP+ astrocytes and **(C)** Cx30 and **(D)** Cx43 (x-axis) and between **(E)** astrocytic radial extent and astrocytic density in hippocampal subregions and layers. A linear regression line has been added to illustrate the associations. Gr, stratum granulare; Mol, stratum moleculare of DG; Or, stratum oriens; Pol, stratum polymorphe; Pyr, stratum pyramidale; Rad, stratum radiatum; SGZ, subgranular zone; SLM, stratum lacunosum moleculare of CA1 and CA3. r, Pearson’s correlation coefficient; *p* < 0.05 significant correlation; *n* = 18 mice.

Both Cx30- and Cx43-Ir correlated positively with the GFAP+ astrocyte density in the different hippocampal layers (Figures 5A, B). The SLM of CA1 and CA3 showed the highest density of GFAP+ astrocytes and the most intense Cx30- (Figure 5A) and Cx43-Ir (Figure 5B). In contrast, the intensity of Cx30- and Cx43-Ir correlated negatively with radial extent of GFAP+ astrocytes (Figures 5C, D). The Pyr and Rad of CA1 and Gr of DG showed the highest radial extend and the lowest Cx30- (Figure 5C) and Cx43-Ir (Figure 5D). Likewise, density and radial extent of GFAP+ astrocytes correlated negatively (Figure 5E). Neither the intensity of C30- and Cx43-Ir nor the density of GFAP+ astrocytes correlated significantly with any of the other morphological parameters (Supplementary Figures 3, 4).

## 4 Discussion

The understanding of the heterogeneity of astrocytes in different brain regions and cortical layers is of great interest, as it contributes decisively to elucidate the multiple roles of astrocytes in supporting neurons in terms of nutrition supply and synaptic plasticity under physiological and pathological conditions (Matyash and Kettenmann, 2010; Zhang and Barres, 2010; Jinno, 2011; Keller et al., 2018; Li et al., 2023; Viana et al., 2023; Karpf et al., 2022). In this study, we provide the first comprehensive and comparative analysis of the spatial distribution of astrocytic density, morphology, and the major astrocytic gap junction proteins Cx30 and Cx43 in the subregions and layers of the mouse hippocampus. Our data show an association between the intensity of the astrocytic gap junction proteins Cx30 and Cx43 and GFAP+ astrocytes and an association between higher astrocyte density and lower radial extent of the GFAP+ processes in hippocampal subregions and layers.

The density of GFAP+ astrocytes was highest in the SLM of the CA1 and CA3, which is consistent with an earlier study showing the highest density of GFAP+ astrocytes in the SLM of the mouse hippocampus (Shimada et al., 1992). In addition, the SLM has the abundance of microvessels (Shimada et al., 1992) and the highest glucose uptake (Shimada et al., 1989) in the mouse hippocampus. These data suggest that in the SLM, a dense network of astrocytes interacting with the dense capillary network is primarily involved in the uptake of metabolites such as glucose (Shimada et al., 1992). The astrocytes in the SLM have comparatively short GFAP+ processes and the lowest radial extent, which is consistent with the study by Viana (Viana et al., 2023) and also with the assumption by Shimada et al. (1992) that the astrocytes in contact with the capillary network tend to have shorter processes. Importantly, the intensity of Cx30- and Cx43-Ir was also highest in the SLM of the CA1 and CA3. Astrocytes are metabolically coupled to each other via Cx30 and Cx43, but also to neurons, in order to provide them with essential metabolites such as glucose and lactate (Mayorquin et al., 2018). Astrocytes also interact with endothelial cells via Cx43 to regulate the permeability of the blood-brain barrier and thus glucose homeostasis (De Bock et al., 2011, 2022; Zhao et al., 2018). However, additional testing of astrocytic gap junction coupling remains to be performed in future studies to confirm functional heterogeneity across the hippocampal layers. Taken together, these data suggest that the uptake and distribution of nutrients from the capillary network in the SLM occurs via the Cx-coupled network of astrocytes that have a small radial extent.

In the DG, there is a similar correlation between the density of astrocytes and microvessels (Shimada et al., 1992), which are significantly higher in Pol and Mol than in Gr (Shimada et al., 1992). Similarly, we found a higher density of GFAP+ astrocytes, a smaller radial extent of GFAP+ processes and a higher intensity of Cx30- and Cx43-Ir in Pol and Mol than in Gr. These data suggest that Cx30 and Cx43 participate in uptake and distribution of nutrients from the capillary network in the Pol and Mol via the Cx-coupled network of small radially extended astrocytes. However, in contrast to previous studies on astrocyte heterogeneity in the mouse hippocampus (Viana et al., 2023; Shimada et al., 1992), we included the SGZ, one of the main neurogenic niches. Here we found the highest density of GFAP+ astrocytes, the highest intensity of Cx30-Ir and a high intensity of Cx43-Ir. In addition to regular astrocytes, GFAP+ cells in SGZ also include radial glial-like neural stem cells that continuously proliferate and produce progenitor cells that differentiate into mature granular neurons that are integrated into hippocampal circuits, thus playing a crucial role in maintaining hippocampal structural and functional plasticity (Ming and Song, 2011). Cx30/43-DKO mice show significant decrease in proliferation of radial glia-like neural stem cells in the SGZ and number of granular neurons, indicating the important role of this Cx in adult neurogenesis (Kunze et al., 2009). In particular, channel-dependent functions of Cx43 are essential for adult neurogenesis in the SGZ (Zhang et al., 2018). Remarkably, the blood vessel density is higher within the SGZ compared to other DG layers (Sun et al., 2015). The blood vessels in the SGZ represent an integral part of the neurogenic niche in the SGZ, providing not only oxygen and nutrients but also hormones and neurotrophic factors that promote self-renewal, proliferation and differentiation of stem cells (Karakatsani et al., 2023). Astrocytes might act as key regulators of blood vessels in the SGZ by modulating blood flow, contributing to vascular stability and growth, and facilitating communication between the vascular system and neural stem cells, thereby preserving the neurogenic niche (Karakatsani et al., 2023). In the other large neuronal stem cell niche, the subventricular zone (SVZ), there is a direct interaction between neural stem cells and endothelial cells, in which Cx43, expressed by both cell types, plays an essential role. The endothelial cells appear to modulate neural stem cell proliferation in a Cx43-dependent channel-independent manner (Genet et al., 2023). Moreover, the astrocytes in the SGZ show the strongest expression of the two main glutamate receptors and the highest glutamate transporter current (Karpf et al., 2022). Taken together these data suggest that Cx30 and Cx43 participate in uptake and distribution of nutrients and signaling molecules from the capillary network in the SGZ via the Cx-coupled network of astrocytes and radial glial-like neural stem cells.

Astrocytes Cx also play a crucial role in hippocampal neuron communication and plasticity (Hösli et al., 2022). In the mouse CA1, Rad has the highest density of total and excitatory synaptic input, while SLM has a higher density of inhibitory input than Rad and Or (Santuy et al., 2020). A study in the human CA1, which in contrast to the aforementioned study also examined the Pyr, showed the highest density of total and excitatory synaptic contacts in the Pyr, followed by Rad and then Or and SLM, while SLM also had the highest density of inhibitory synapses (Montero-Crespo et al., 2020). Since we observe the highest density of GFAP+ astrocytes and the most intense Cx30 and Cx43-Ir in the SLM, there is therefore no association with the density of excitatory synapses, but if anything, with the density of inhibitory synapses. Although some studies suggest that Cx30 and Cx43 are more strongly associated with excitatory than inhibitory synaptic transmission (Pannasch et al., 2014), other reports suggest that astrocytes are active modulators of both excitatory and inhibitory synapses in the mouse hippocampus via Cx (Hardy et al., 2021), and that astrocyte networks have a physiologically optimized size to adequately regulate neuronal functions (Hardy et al., 2023). However, it is likely that the Cx-mediated morphological changes of the fine astrocyte processes, that envelop the synapses and thus modulate synaptic strength and plasticity, play a greater role in excitatory synapses than in inhibitory synapses (Pannasch et al., 2014). Although GFAP staining is not suitable for visualizing these fine synapse-associated astrocytic processes, it can be used to show the backbone structure of the processes and, thus, the morphological complexity (Viana et al., 2023). Moreover, GFAP is a component of the intermediate filament cytoskeleton, which not only ensures the structural integrity and spatial organization of the fine astrocyte processes but is also involved in the transport of organelles (Creighton et al., 2019; Jones et al., 2018, Pajares et al., 2023). Regarding the morphology of the GFAP+ processes in CA1 and CA3, we report that Rad and Pyr showed the highest radial extent, the highest number of total and first order processes (indicated by number of trees), and the longest processes, indicating the most complex and far-reaching morphology in these two layers, which have the highest density of excitatory synapses (Santuy et al., 2020; Montero-Crespo et al., 2020). Our findings largely align with those of Viana et al., 2023, which also show the highest complexity of the GFAP+ processes in the Rad of CA1, but did not investigate the Pyr or CA3. Therefore, we assume that the large radial extension of GFAP+ astrocytes in the CA1 and CA3 layers with high synapse density is related to the transport of organelles such as mitochondria and endoplasmic reticulum, which are essential for maintaining ion and neurotransmitter homeostasis.

Importantly, the astrocytes are able to modulate their morphology to build slightly overlapping individual territories that adapt available space, which known as “tiling,” resulting in an inverse relationship between the astrocyte density and their extent (domain size) (Grosche et al., 2013; Barber et al., 2021). Among all hippocampal layers investigated in this study, the astrocyte density was positively correlated with the intensity of Cx30- and Cx43-Ir and negatively correlated with the radial extent of GFAP+ processes. Consistently, intensities of Cx30- and Cx43-Ir were negatively correlated with the radial extent of GFAP+ processes. This suggests an inverse relationship between the number of astrocytes/astrocytic gap junctions and the size of the area covered by their backbone within the hippocampal layers. Noteworthy, the negative correlation between Cx-Ir may be an indirect reflection of the negative correlation between astrocytic density and their radial extent.

Our study leads to two major conclusions regarding the diversity of astrocytes in the subregions and layers of the hippocampus. (1) The highest density of GFAP+ astrocytes, the smallest radial extension of GFAP+ processes, and the highest density of Cx30 and Cx43 are found in the layers where, according to the literature, the most microvessels are located. This includes the SLM and also SGZ, which, as a neurogenic niche, exhibits a high metabolic rate. We therefore suspect a connection between these astrocytic properties and the uptake of nutrients from the capillaries. (2) The largest radial extension of GFAP+ processes is found in the CA layers, which are known to have the highest synapse density. We suspect that this large backbone, on the one hand, serves as a scaffold for the fine processes and, on the other hand, for the transport of cell organelles into synaptic areas. Future studies should test these proposed relationships and investigate the dynamic changes in these astrocytic properties under physiological and pathological conditions.

### 4.1 Limitations

In this study, we used GFAP as an astrocyte marker, which also detects neural stem/progenitor cells and specialized glia and has also been used in relevant studies (e.g., Kunze et al., 2009; Shimada et al., 1992; Viana et al., 2023), ensuring good comparability. However, we cannot rule out the possibility that we have not detected all subpopulations of astrocytes.

## Data Availability

The datasets presented in this study can be found in online repositories. The names of the repository/repositories and accession number(s) can be found below: https://researchdata.hhu.de/handle/entry/199.

## Supplementary material

Supplementary Figure 1 Representative photomicrograph showing GFAP-immunoreactive (Ir) astrocytes in hippocampal regions. Analysis involved stratum oriens (Or), stratum pyramidale (Pyr), stratum radiatum (Rad), and stratum lacunosum moleculare (SLM) of CA1 and CA3, as well as stratum polymorphe (Pol), stratum moleculare (Mol), stratum granulare (Gr), and the subgranular zone (SGZ), defined as a two-nucleus-wide area along the inner border of Gr, of the DG. Scale bar = 200 μm.

Supplementary Figure 2 Heterogeneity of astrocytic morphology in mouse in the hippocampal layers. Quantification of astrocyte morphology in **(A–C)** in CA1, **(D–F)** in CA3 and **(G–I)** in DG. Gr, stratum granulare; Mol, stratum moleculare of DG; Or, stratum oriens; Pol, stratum polymorphe; Pyr, stratum pyramidale; Rad, stratum radiatum; SGZ, subgranular zone; SLM, stratum moleculare of CA1 and CA3. One-way ANOVA followed by Tukey *post-hoc* tests. *n* = 10 in DG and *n* = 18 mice in CA1 and CA3. **p* < 0.05; ***p* < 0.01; ****p* < 0.001, *****p* < 0.0001.

Supplementary Figure 3 Scatterplots showing the relationship between astrocytic Cx30 and Cx43 (*x*-axis) with different morphological parameters of astrocytes (*y*-axis) in hippocampal subregions. A simple linear regression line has been added to illustrate the correlation. Gr, stratum granulare; Mol, stratum moleculare of DG; Or, stratum oriens; Pol, stratum polymorphe; Pyr, stratum pyramidale; Rad, stratum radiatum; SGZ, subgranular zone; SLM, stratum moleculare of CA1 and CA3. (r) indicates the correlation coefficient using Pearson correlation test. *p* < 0.05 indicates a significant correlation.

Supplementary Figure 4 Scatterplots showing the relationship between astrocyte density/mm^2^ (*x*-axis) with different morphological parameters of astrocytes (*y*-axis) in hippocampal subregions. A simple linear regression line has been added to illustrate the correlation. Gr, stratum granulare; Mol, stratum moleculare of DG; Or, stratum oriens; Pol, stratum polymorphe; Pyr, stratum pyramidale; Rad, stratum radiatum; SGZ, subgranular zone; SLM, stratum moleculare of CA1 and CA3. (r) indicates the correlation coefficient using Pearson correlation test. *p* < 0.05 indicates a significant correlation.
