# Supplementary figures and images for: Heterogeneity of astrocyte density, morphology and connexins in the mouse hippocampus
Source: Front Neuroanat. 2026 Mar 9;20:1771439. doi: 10.3389/fnana.2026.1771439 (PMC13006667; doi:10.3389/fnana.2026.1771439)

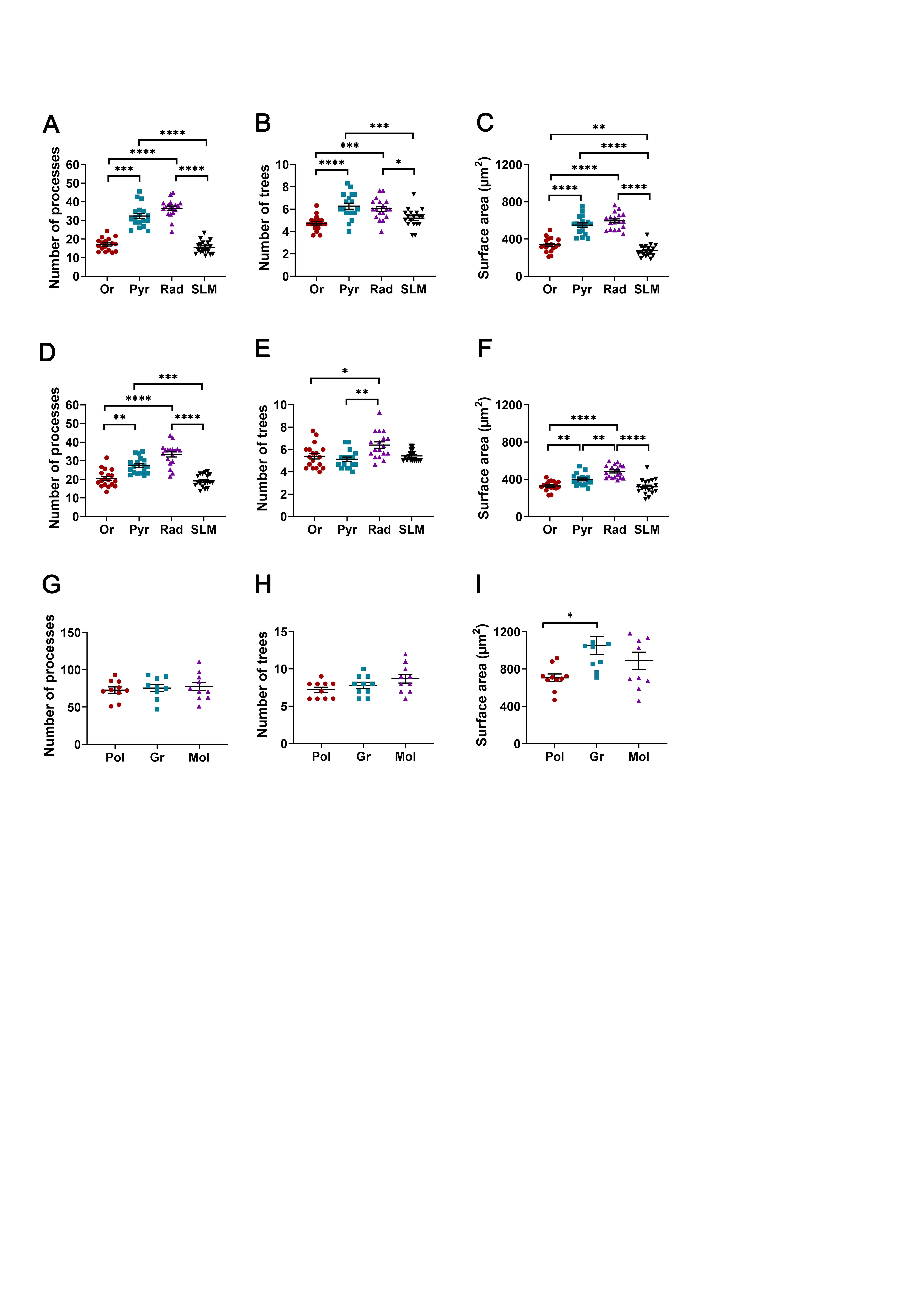

Supplement: Supplementary Figure 2 — Heterogeneity of astrocytic morphology in mouse in the hippocampal layers. Quantification of astrocyte morphology in (A–C) in CA1, (D–F) in CA3 and (G–I) in DG. Gr, stratum granulare; Mol, stratum moleculare of DG; Or, stratum oriens; Pol, stratum polymorphe; Pyr, stratum pyramidale; Rad, stratum radiatum; SGZ, subgranular zone; SLM, stratum moleculare of CA1 and CA3. One-way ANOVA followed by Tukey post-hoc tests. n = 10 in DG and n = 18 mice in CA1 and CA3. *p < 0.05; **p < 0.01; ***p < 0.001, ****p < 0.0001. [file Image_2.jpeg]

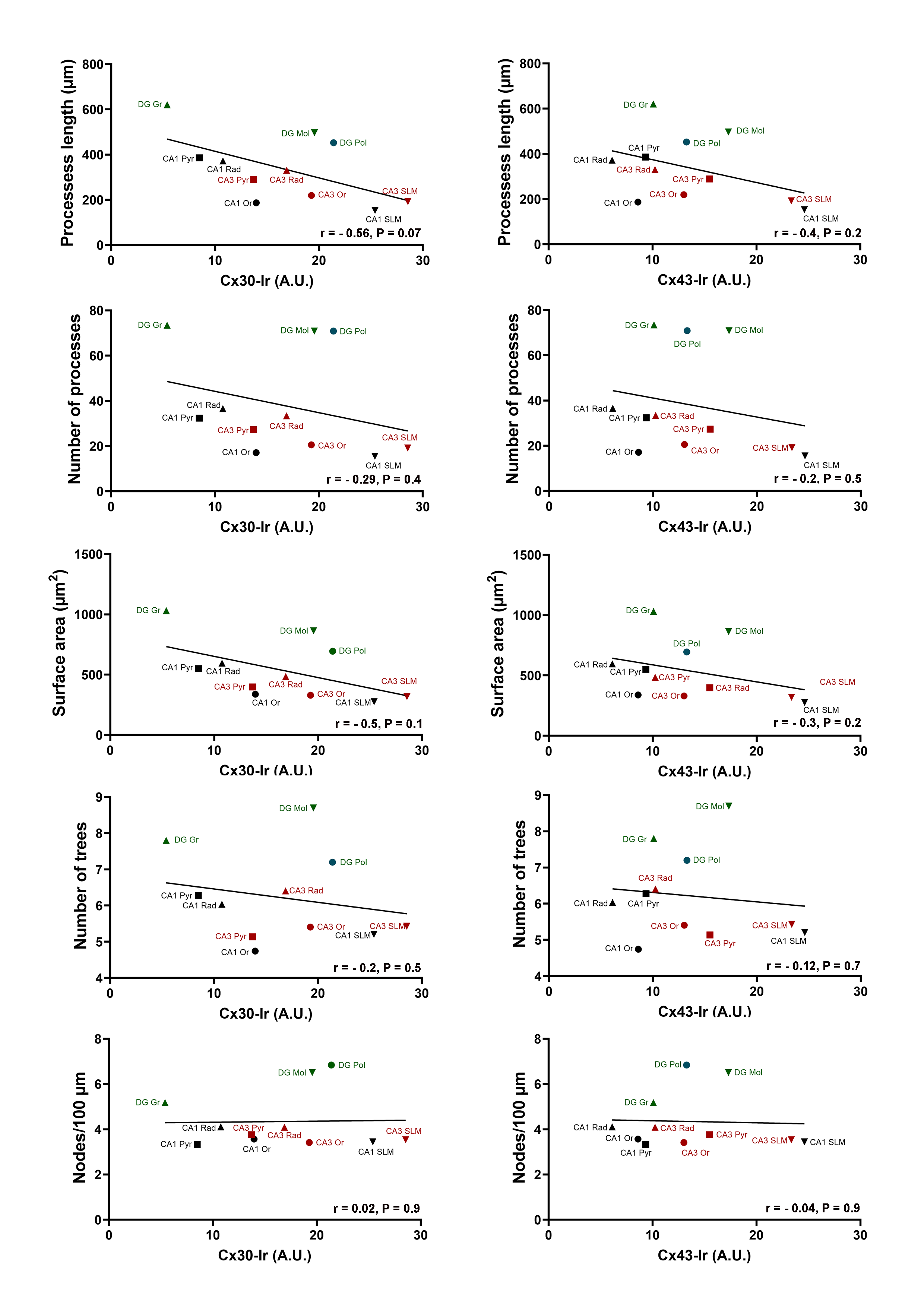

Supplement: Supplementary Figure 3 — Scatterplots showing the relationship between astrocytic Cx30 and Cx43 (x-axis) with different morphological parameters of astrocytes (y-axis) in hippocampal subregions. A simple linear regression line has been added to illustrate the correlation. Gr, stratum granulare; Mol, stratum moleculare of DG; Or, stratum oriens; Pol, stratum polymorphe; Pyr, stratum pyramidale; Rad, stratum radiatum; SGZ, subgranular zone; SLM, stratum moleculare of CA1 and CA3. (r) indicates the correlation coefficient using Pearson correlation test. p < 0.05 indicates a significant correlation. [file Image_3.tif]

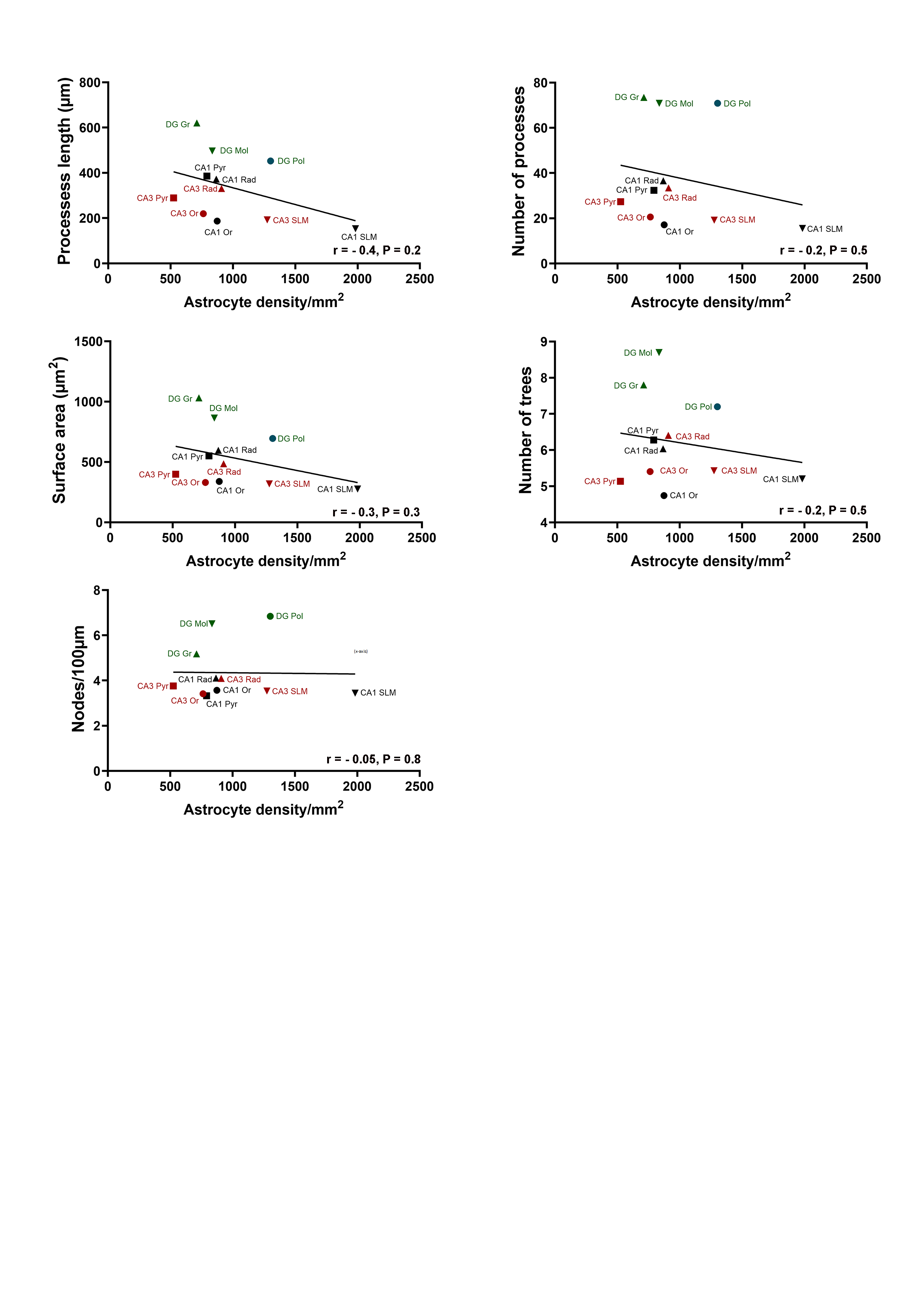

Supplement: Supplementary Figure 4 — Scatterplots showing the relationship between astrocyte density/mm2 (x-axis) with different morphological parameters of astrocytes (y-axis) in hippocampal subregions. A simple linear regression line has been added to illustrate the correlation. Gr, stratum granulare; Mol, stratum moleculare of DG; Or, stratum oriens; Pol, stratum polymorphe; Pyr, stratum pyramidale; Rad, stratum radiatum; SGZ, subgranular zone; SLM, stratum moleculare of CA1 and CA3. (r) indicates the correlation coefficient using Pearson correlation test. p < 0.05 indicates a significant correlation. [file Image_4.tif]
